# Effects of an Immersive Virtual Reality–Based Exercise Intervention on Psychological and Physiological Outcomes in College Students: Randomized Controlled Trial

**DOI:** 10.2196/75777

**Published:** 2025-12-15

**Authors:** Wenxi Liu, Daniel J McDonough, John Oginni, Jessh Mavoungou, Zan Gao

**Affiliations:** 1 Department of Physical Education Shanghai Jiao Tong University Shanghai, Shanghai China; 2 School of Public Health University of Minnesota-Twin Cities Minneapolis, MN United States; 3 Department of Kinesiology, Recreation, and Sport Studies University of Tennessee at Knoxville Knoxville, TN United States

**Keywords:** body composition, cardiovascular fitness, depression, health, mood, motivation

## Abstract

**Background:**

Physical inactivity is a major public health issue among college students, often exacerbated by academic pressures and lifestyle shifts. Traditional exercise interventions often face challenges with adherence due to low motivation and engagement. Immersive virtual reality (VR)–based exercise interventions may address these barriers by providing interactive and motivating experiences, yet empirical evidence regarding their psychological and physiological benefits remains scarce.

**Objective:**

This study aims to evaluate the effects of a 4-week immersive VR-based exercise intervention on psychological and physiological health outcomes in college students.

**Methods:**

A randomized controlled trial was conducted involving 36 college students randomized into either a VR exercise group (n=17, 47%) or a no-intervention control group (n=19, 53%). Participants in the VR group engaged in immersive VR cycling sessions (two 60-minute sessions weekly) using the VirZoom VR system, while the control group continued their normal routines. Psychological outcomes were assessed during preintervention and postintervention assessments, including exercise motivation (Behavioral Regulation in Exercise Questionnaire-2), mood states (Brunel Mood Scale), and depressive symptoms (Beck Depression Inventory). Physiological outcomes assessed were cardiovascular fitness (3-Minute Step Test) and body composition (bioelectrical impedance analysis). We used a 2-way repeated measures ANOVA to analyze the effects of the intervention.

**Results:**

Significant time×group interactions indicated enhanced intrinsic motivation (*P*=.02; η^2^=0.25); improved mood states with increased vigor (*P*=.01; η^2^=0.18); and decreased confusion (*P*=.01; η^2^=0.17), fatigue (*P*=.02; η^2^=0.16), and tension (*P*=.003; η^2^=0.24) in the VR group. Depressive symptoms were also significantly reduced (*P*=.03; η^2^=0.14). Physiological outcomes showed significant improvements in the VR group, including decreased body fat percentage (*P*<.001; η^2^=0.34) and enhanced cardiovascular fitness (*P*<.001; η^2^=0.47) compared to the control group.

**Conclusions:**

This study indicated that a 4-week immersive VR-based exercise intervention may confer short-term psychological and physiological benefits among college students compared to the no-intervention control group under COVID-19 pandemic–related constraints. Future studies should adopt active control designs and be conducted in real-world settings, incorporating objectively determined intensity monitoring and follow-up to further investigate effectiveness and real-world scalability.

**Trial Registration:**

ClinicalTrials.gov NCT06902727; https://clinicaltrials.gov/study/NCT06902727

## Introduction

Physical inactivity has emerged as a major public health issue worldwide, linked to an increased risk of chronic diseases, such as obesity, cardiovascular diseases, and mental health disorders [1]. Despite widespread awareness of these risks, a significant portion of the global population remains inactive [2]. In particular, college students face unique challenges that contribute to physical inactivity, including the demands of academic life, transitioning to independent living, and balancing work and social commitments [3]. Research highlights a steep decline in physical activity (PA) levels among this population, with fewer than half meeting the American College of Sports Medicine–recommended guidelines of 150 minutes of moderate to vigorous PA weekly [4]. Addressing physical inactivity in this population is a public health priority, yet existing interventions have had limited success in sustaining long-term engagement [5]. The ongoing challenges of stress and mental health in higher education further underscore the need for interventions that promote both physical and mental well-being [6-8].

Virtual reality (VR)–based exercise represents a promising solution. By combining physical exertion with immersive and interactive virtual environments, VR-based exercise addresses common barriers to PA, such as monotony and a lack of motivation [9-11]. Immersive VR systems offer unique features that enhance the user experience, including customizable challenges, real-time feedback, and an increased sense of “presence,” the feeling of “being there” in a virtual environment [12,13]. These elements not only increase enjoyment but also reduce perceived exertion and discomfort, facilitating greater adherence to exercise programs [14-16].

From a psychological perspective, VR aligns with the principles of self-determination theory (SDT), which emphasizes the importance of autonomy, competence, and relatedness in sustaining behavior change [17-19]. VR-based exercise can enhance intrinsic motivation by providing users with choices (autonomy), offering achievable challenges and performance feedback (competence), and creating opportunities for social interaction in multiplayer environments (relatedness), fostering a sense of personal accomplishment and enjoyment [20-22]. Moreover, VR-based exercise has demonstrated potential for improving mental health outcomes, including mood enhancement and reductions in depressive symptoms [10,23].

Despite the promising findings, high-quality experimental evidence on the effectiveness of VR-based exercise interventions in healthy populations remains limited. Previous studies have primarily focused on acute effects or clinical populations, leaving significant gaps in understanding the broader applicability of VR-based exercise [10,24-26]. Therefore, the purpose of this study was to examine the effects of a 4-week VR-based exercise intervention on health-related psychological (ie, motivation, mood, and depressive symptoms) and physiological (ie, body composition and cardiovascular fitness) outcomes among college students. This study hypothesized that intervention participants would have significant improvement in health-related psychological and physiological outcomes compared to a no-intervention control group after a 4-week VR-based exercise intervention. The findings of this study would provide insights into the potential of VR-based exercise to improve health-related outcomes in the young adult population.

## Methods

### Study Design

This study used a 2-arm randomized controlled trial to evaluate the efficacy of a 4-week VR-based exercise intervention on health-related psychological and physiological outcomes in college students. The use of a no-intervention control group was chosen to first establish the efficacy of this specific VR intervention before comparing it to other forms of PA. Participants were randomly assigned to either the intervention group or the control group, with assessments conducted at baseline and after the intervention (4 weeks).

### Participants

Participants were recruited from a single large public university in the Midwestern United States via university-wide email newsletters and departmental listserves, targeting both undergraduate and graduate students. Recruitment and data collection took place between August 2020 and March 2021. We determined the required sample size using G*Power (version 3.1; Heinrich Heine University Düsseldorf) for a repeated measures ANOVA with a between-factor interaction. With an effect size f(V) of 0.25 (a medium effect), an α of .05, and power of 0.80, with 2 groups and 2 measurements and a correlation among repeated measures of 0.5, the required total sample size was 34. Our final analyzed sample of 36 exceeded this requirement.

The inclusion criteria were as follows: (1) college students aged between 18 and 35 years, (2) without diagnosed physical or mental disabilities, (3) willing to provide informed consent and complete the Physical Activity Readiness Questionnaire in English, and (4) no self-reported motion sickness symptoms during a brief VR familiarization and screening session. Participants were also screened for any musculoskeletal issues that would contraindicate participation in this study. Information on previous exercise experience was also collected.

### Ethical Considerations

This study was approved by the institutional review board of the University of Minnesota Twin Cities (00010580). All procedures were conducted in accordance with the ethical standards of the institutional research committee and the Declaration of Helsinki 1964 and its later amendments. All participants provided written informed consent before participating in this study. The consent process included a detailed explanation of the study procedures, potential risks and benefits, the right to withdraw at any time without penalty, and contact information for the research team and the institutional review board. All study data were deidentified and assigned unique participant ID numbers. Data were stored on password-protected computers accessible only to the research team. Hard copies of consent forms were stored separately from study data in locked filing cabinets. In addition, participants received a US $20 incentive gift card upon completing all assessments (details in Multimedia Appendices 1-4). This trial was registered on ClinicalTrials.gov (NCT06902727).

### Measurements

#### Motion Sickness

Participants were asked to complete a 2-item survey on a 3-point Likert scale (1=not at all; 3=a lot) for assessing the symptoms of motion sickness. Two questions were included in the survey: (1) “When riding the VR bike, I feel sick to my stomach” (2) “When riding the VR bike, I feel dizzy.” The survey was administered during the preintervention screening session.

#### Demographics and Anthropometrics

Participants’ age, race or ethnicity, and sex were obtained via baseline self-administered surveys. Participants’ height was measured using the Seca stadiometer. Body weight and body fat (BF) percentage were measured by the Tanita BC-558 IRONMAN Segmental Body Composition Monitor. In addition, participants’ previous VR experiences were assessed at baseline.

#### PA Assessment

Participants’ free-living PA levels were assessed via the International Physical Activity Questionnaire (IPAQ) Short Form. The IPAQ Short Form assessed 3 specific types of activity: walking, moderate-intensity activities, and vigorous-intensity activities. Participants were asked to recall the time spent (in minutes) walking, performing moderate-intensity PA, and engaging in vigorous-intensity PA in the past 7 days. Regarding the IPAQ scoring, the weekly PA volume was computed by weighing each type of activity by its energy requirements defined in metabolic equivalents (METs) to yield a score in MET-minutes. The mean weekly PA levels were calculated as the study outcomes to examine the difference between the 2 groups at baseline.

#### Exercise Motivation

Participants’ general exercise motivation was assessed by the Behavioral Regulation in Exercise Questionnaire-2 (BREQ-2) [27]. The BREQ-2 has 19 items assessing the continuum of behavioral regulation in exercise, which includes amotivation, external regulation, introjected regulation, identified regulation, and intrinsic regulation. The BREQ-2 has demonstrated good reliability and validity in previous research.

#### Mood States

Participants’ mood states were measured using the Brunel Mood Scale (BRUMS) [28]. The BRUMS assesses mood across 6 subscales (anger, confusion, depression, fatigue, tension, and vigor) using a 24-item questionnaire, with responses ranging from 0 (not at all) to 4 (extremely). The BRUMS has shown good psychometric properties in adult populations.

#### Depressive Symptoms

The Beck Depression Inventory was used to assess participants’ depressive symptoms [29]. This is a 21-item, self-report rating inventory that measures characteristic attitudes and symptoms of depression. The total score was calculated to evaluate the severity of depressive symptoms.

#### Cardiovascular Fitness

Participants’ cardiovascular fitness (CRF) was assessed by the 3-Minute Step Test [30]. Participants stepped on and off a 30-cm plyometric box in time with a metronome set to 96 bpm for 3 minutes. Heart rate was measured immediately after the test via Omron Fingertip Oximeter. The change in heart rate from baseline to after the test served as the primary outcome for cardiovascular fitness.

### Procedure

Recruitment was conducted via online channels, including university email lists and departmental newsletters. Interested college students contacted the investigator to schedule a preintervention screening session. To mitigate motion sickness risk, potential participants underwent a 15-minute VR biking session using the VirZoom VR bike system during which they engaged in both “Le Tour” and “Race Car” exergames. After the session, participants completed the 2-item motion sickness survey. Only those who completed the session without reporting motion sickness symptoms were enrolled in the study. Enrolled participants completed demographic surveys (age, sex, ethnicity, and previous VR experience) and underwent physiological and psychological assessments, including height, weight, body composition (BF percentage and BMI), CRF (step test), exercise motivation (BREQ-2), mood (BRUMS), and depressive symptoms (Beck Depression Inventory). Following baseline assessments, participants were randomized into the intervention or control group. The intervention group engaged in VR-based exercise sessions twice weekly for 4 weeks, while the control group maintained their usual activities. After 4 weeks, all participants returned for postintervention assessments identical to the baseline measures.

### Randomization

Randomization was executed using a computer-generated sequence in Microsoft Excel, allocating participants in a 1:1 ratio to either the intervention or control group. This process ensured equal distribution of participants across both groups, minimizing selection bias.

### Intervention Group

**Figure 1:**
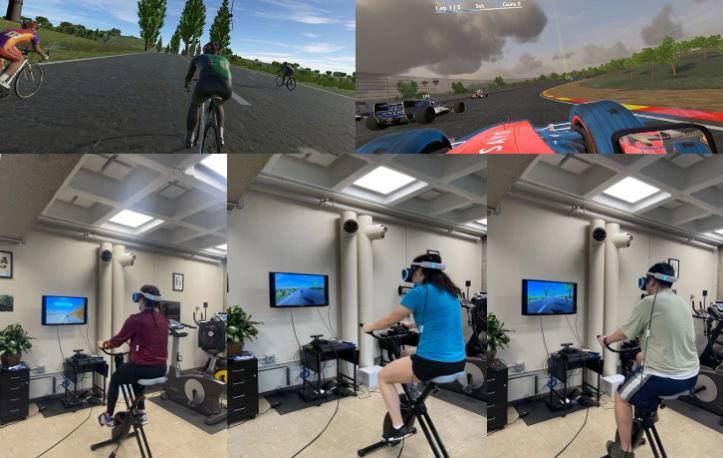
Participants engaging in immersive virtual reality–based exercise intervention using the VirZoom VR exercise bike connected to the PlayStation VR system.

Participants in the intervention group engaged in an immersive VR cycling regimen using the commercial VirZoom VR exercise bike connected to a PlayStation VR system (Sony), which includes a head-mounted display. The intervention consisted of exercising for 1 hour per session, twice weekly, for 4 weeks. Each session included 2 VR exergames (demonstrated in Figure 1). The first game was “Le Tour,” a cycling race game through a virtual scenic mountain road featuring 15 timing gates. Participants aimed to meet various performance goals at each gate, such as maintaining specific pedaling speeds or drafting behind virtual cyclists. The game offered customizable workout plans with 2 intensity modes—comfort (light to moderate) and intense (moderate to vigorous). Participants were encouraged to exercise at a moderate-intensity level, and perceived exertion was monitored. After 30 minutes of gameplay, the system automatically transitioned participants to the second game to maintain engagement and adherence. The second game was “Race Car,” a high-speed racing game where participants pedal to control a virtual race car, aiming to surpass all virtual competitors. The game features a “ghost car” representing the fastest previous performance, encouraging participants to beat their own or others’ records. Our previous study demonstrated that VirZoom VR cycling effectively elicited moderate to vigorous PA, with most of the exercise duration classified as vigorous intensity [11,20,31].

### Control Group

Participants in the control group were instructed to maintain their usual routine and daily activities, without engaging in any other PA interventions during the 4-week study period. We did not formally measure the PA levels of the control group throughout the study. Both groups received identical assessments at baseline and after intervention.

### Intervention Fidelity

To ensure intervention fidelity, several strategies were implemented, including providing detailed instructions on survey completion, conducting preintervention tutorials and hands-on VR exercise sessions, and tracking attendance and adherence through exercise session logs. A Microsoft Excel–based tracking system reminded participants of upcoming assessments, while individualized schedules and email reminders were sent to intervention participants.

### Statistical Analysis

Data were analyzed using SPSS Statistics (version 27.0; IBM Corp). Descriptive statistics (means, SDs, and frequencies) were calculated for demographic variables, physiological measures, and psychological outcomes. Baseline differences between the intervention and control groups were evaluated using independent 2-tailed *t* tests for continuous variables and chi-square tests for categorical variables to confirm effective randomization. A robust regression–based outlier detection procedure was used to identify outliers, and any identified outliers were excluded from the analyses. A 2-way repeated measures ANOVA was used to examine the changes in the following health-related physiological and psychological outcomes: exercise motivation (amotivation, external regulation, introjected regulation, identified regulation, and intrinsic regulation), mood states (anger, confusion, depression, fatigue, tension, and vigor), depressive symptoms (total depression score), and physiological measures (BF percentage and CRF).

## Results

### Participant Flow

**Figure 2:**
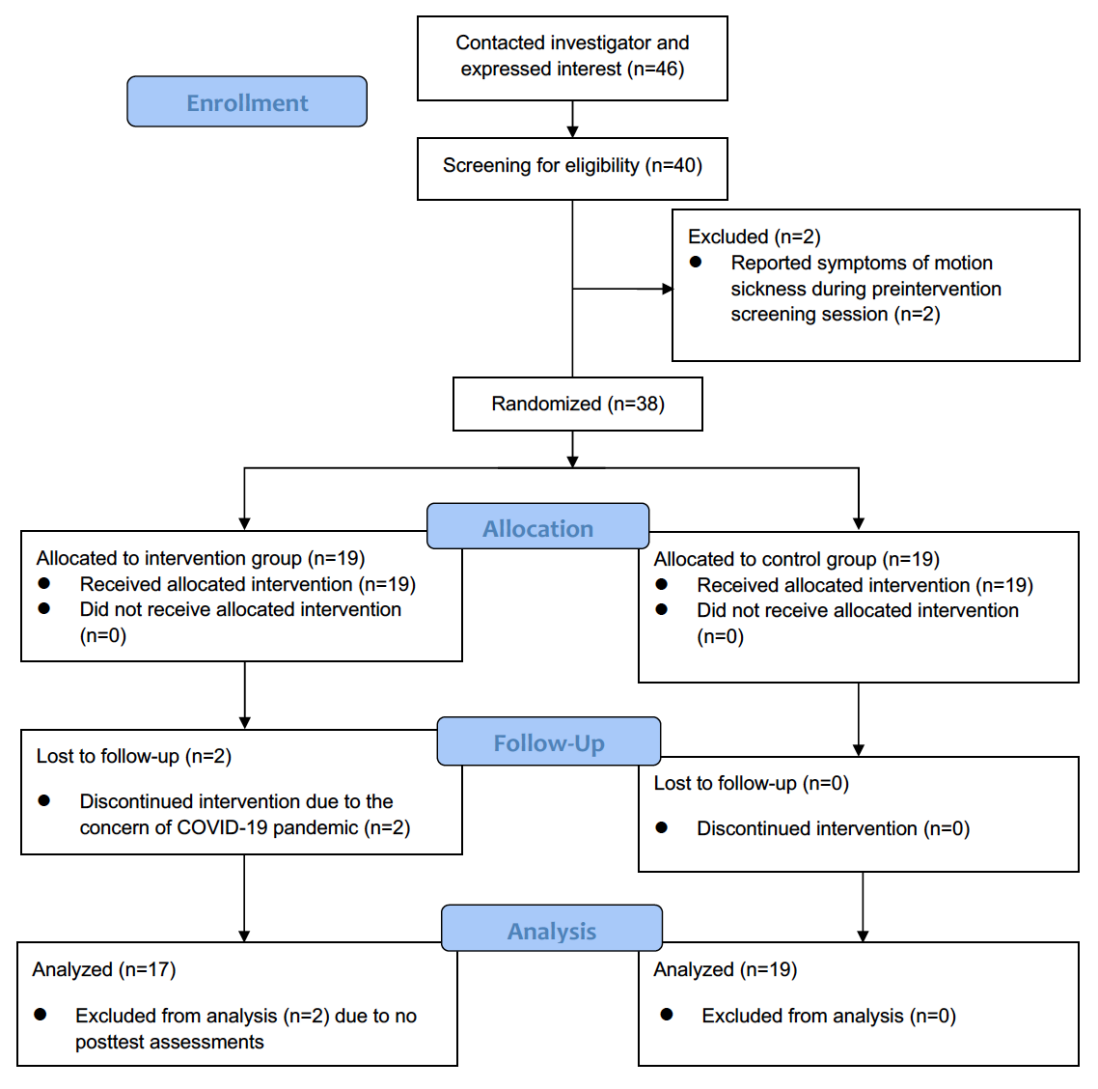
Consolidated Standards of Reporting Trials (CONSORT) flow diagram of participant flow.

Figure 2 demonstrates the participant flow of this study. At the beginning, 46 college students contacted the principal investigator and expressed interest in participation via email. Next, 40 (87%) students came to the laboratory for the prestudy screening session. College students who completed the screening session and reported no motion sickness symptoms were eligible for this study. In total, 2 (5%) college students reported motion sickness symptoms and thus were excluded from this study. As a result, 38 (95%) eligible college students were enrolled in the study and randomized into intervention and control groups. Notably, 2 (5%) participants dropped out of the intervention group during the first week of intervention, expressing concerns about participating in an in-person study during the COVID-19 pandemic. Finally, 36 participants (intervention group: n=17, 47%; control group: n=19, 53%) completed baseline and postintervention assessments. Their data were entered for statistical analysis.

### Baseline Participant Characteristics

The descriptive statistics of participants’ baseline demographic, physiological, and psychological outcomes by group are shown in Table 1. The chi-square test and the independent *t* test indicated that there were no statistically significant differences in demographic, physiological, and psychological characteristics between the intervention and control groups at baseline. Regarding previous VR experience, 44.4% (16/36) of the participants reported having previous VR experience, and 55.6% (20/36)reported no previous VR experience. The chi-square test of VR experience between 2 groups indicated there was no statistically significant group difference at baseline.

**Table 1:** Baseline group comparisons for study outcomes.

Characteristic			Intervention (n=17)	Control (n=19)		*P* value
**Sex, n (%)**						.54
	Male	8 (47)		7 (37)		
	Female	9 (53)		12 (63)		
**Race or ethnicity, n (%)**						.41
	Asian	10 (59)		10 (53)		
	White	6 (35)		5 (26)		
	Other	1 (6)		4 (21)		
Age (y), mean (SD)			22.82 (2.51)	24.84 (4.14)		.09
Height (cm), mean (SD)			171.26 (7.21)	169.71 (7.35)		.53
Weight (kg), mean (SD)			74.38 (16.49)	73.56 (14.75)		.88
BMI (kg/m^2^), mean (SD)			25.21 (4.36)	25.37 (3.69)		.91
Body fat percentage, mean (SD)			26.11 (7.14)	27.65 (5.18)		.46
Cardiovascular fitness (bpm), mean (SD)			113 (22.10)	115.95 (16.64)		.65
Physical activity (metabolic equivalent min/wk)			1152.06 (618.57)	1080.26 (935.04)		.79
**Self-determination theory–** **based motivation, mean (SD)**						
	Amotivation	1.57 (0.64)		1.75 (0.64)	.41	
	External regulation	1.94 (0.71)		2.32 (0.86)	.17	
	Introjected regulation	3.33 (1.14)		2.86 (0.88)	.17	
	Identified regulation	4.00 (0.71)		3.74 (0.65)	.25	
	Intrinsic regulation	3.71 (0.94)		3.63 (0.86)	.81	
**Mood states, mean (SD)**						
	Anger	1.28 (0.57)		1.60 (1.09)	.30	
	Confusion	1.53 (0.75)		1.67 (0.97)	.63	
	Depression	1.41 (0.69)		1.62 (0.81)	.42	
	Fatigue	2.00 (0.54)		2.09 (1.01)	.74	
	Tension	1.85 (0.72)		1.84 (1.03)	.97	
	Vigor	2.74 (0.50)		2.51 (0.74)	.30	
Depression score, mean (SD)			5.94 (5.68)	6.68 (6.25)		.71

### Psychological Outcomes

The descriptive statistics for exercise motivation by group at baseline and 4 weeks are presented in Table 2. Regarding the 5 subscales of motivational regulation, the 2-way repeated measures ANOVA indicated significant main effect of time on amotivation (*F*_1,34_=6.10; *P*=.02; η²=0.15), external regulation (*F*_1,34_=4.37; *P*=.04; η²=0.11), identified regulation (*F*_1,34_=6.55; *P*=.02; η²=0.16), and intrinsic regulation (*F*_1,34_=18.11; *P*<.001; η²=0.35). The significant time×group interactions were observed on identified regulation (*F*_1,34_=6.55; *P*=.02; η²=0.16) and intrinsic regulation (*F*_1,34_=11.21; *P*=.02; η²=0.25). Post hoc analyses further indicated that intervention participants’ identified regulation and intrinsic regulation significantly increased after the 4-week intervention compared to the control group.

The descriptive statistics for mood states are presented in Table 3. Regarding the 6 subscales of mood states, the 2-way repeated measures ANOVA indicated a significant main effect of time on anger (*F*_1,34_=4.54; *P*=.04; η²=0.12), fatigue (*F*_1,34_=5.35; *P*=.03; η²=0.14), and vigor (*F*_1,34_=50.79; *P*<.001; η²=0.6). The significant time×group interactions were observed on confusion (*F*_1,34_=6.72; *P*=.01; η²=0.17), fatigue (*F*_1,34_=6.46; *P*=.02; η²=0.16), tension (*F*_1,34_=10.44; *P*=.003; η²=0.24), and vigor (*F*_1,34_=7.22; *P*=.01; η²=0.18). Our data further indicated that intervention participants perceived less sense of confusion, fatigue, tension, and greater vigor compared to the control group at postintervention assessment.

For depressive symptoms, the results revealed a significant time×group interaction on the mean scores of depression symptom assessment (*F*_1,34_=5.53; *P*=.03; η²=0.14). In the intervention group, the mean total depression score decreased from 5.94 (SD 5.68) at baseline to 3.24 (SD 4.28) after 4 weeks (*P*=.03). In contrast, in the control group, the mean total depression score increased from 6.68 (SD 6.25) at baseline to 7.21 (SD 6.75) after 4 weeks (*P*=.03).

**Table 2:** Descriptive statistics for self-determination theory–based exercise regulations by group.

		Baseline, mean (SD)	After 4 weeks, mean (SD)	*P* value	
**Amotivation**					.05
	Intervention	1.57 (0.64)	1.07 (0.15)		
	Control	1.75 (0.64)	1.61 (0.89)		
**External regulation**					.21
	Intervention	1.94 (0.71)	1.76 (0.56)		
	Control	2.32 (0.86)	1.99 (0.91)		
**Introjected regulation**					.06
	Intervention	3.33 (1.14)	3.67 (0.99)		
	Control	2.86 (0.88)	3.05 (0.79)		
**Identified regulation**					.02
	Intervention	4.00 (0.71)	4.59 (0.33)		
	Control	3.74 (0.65)	3.74 (0.61)		
**Intrinsic regulation**					.02
	Intervention	3.71 (0.94)	4.81 (0.26)		
	Control	3.63 (0.86)	3.76 (0.61)		

**Table 3:** Descriptive statistics for mood subscales by group.

Mood subscale and group		Baseline, mean (SD)	After 4 weeks, mean (SD)	*P* value	
**Anger**					.26
	Intervention	1.28 (0.57)	1.01 (0.06)		
	Control	1.59 (1.09)	1.51 (0.95)		
**Confusion**					.01
	Intervention	1.53 (0.75)	1.15 (0.18)		
	Control	1.67 (0.97)	1.84 (0.97)		
**Depression**					.06
	Intervention	1.41 (0.69)	1.01 (0.06)		
	Control	1.62 (0.81)	1.63 (0.87)		
**Fatigue**					.02
	Intervention	2.00 (0.54)	1.44 (0.40)		
	Control	2.09 (1.01)	2.12 (1.01)		
**Tension**					.003
	Intervention	1.85 (0.72)	1.35 (0.52)		
	Control	1.84 (1.03)	2.00 (1.11)		
**Vigor**					.01
	Intervention	2.74 (0.50)	4.01 (0.50)		
	Control	2.51 (0.74)	3.09 (0.88)		

### Physiological Outcomes

The descriptive statistics for BMI, BF percentage, and CRF by group at baseline and 4 weeks are presented in Table 4. The results indicated that there was a significant main effect of time on CRF (*F*_1,34_=20.65; *P*<.001; η²=0.38). However, there was no significant main effect of time on BMI (*F*_1,34_=1.48; *P*=.23; η^2^=0.04) and BF percentage (*F*_1,34_=3.07; *P*=.09; η²=0.08). In addition, significant time×group interactions were observed on BF percentage (*F*_1,34_=17.26; *P*<.001; η²=0.34) and CRF (*F*_1,34_=30.05; *P*<.001; η²=0.47). Post hoc analyses further indicated that intervention participants’ BF percentage and CRF had significant improvement as compared to the control group at the postintervention assessment.

**Table 4:** Descriptive statistics for physiological outcomes by group.

Physiological outcomes			Baseline, mean (SD)		After 4 weeks, mean (SD)	*P* value	
**BMI (kg/m^2^)**							.55
	Intervention	25.21 (4.36)		25.00 (4.05)			
	Control	25.52 (3.71)		24.87 (5.11)			
**Body fat percentage**							<.001
	Intervention	26.11 (7.14)		24.51 (6.98)			
	Control	27.28 (5.09)		28.16 (4.60)			
**Cardiovascular fitness (bpm)**							<.001
	Intervention	113 (22.10)		96.12 (16.48)			
	Control	115.19 (16.64)		117.53 (16.56)			

## Discussion

### Principal Findings

This study demonstrated that a 4-week immersive VR exercise program significantly improved certain psychological and physiological outcomes among college students. Participants in the VR group reported higher intrinsic and identified motivation, more positive mood states, and fewer depressive symptoms while also achieving measurable reductions in BF percentage and better CRF compared with the control group. Overall, the VR-based exercise intervention produced meaningful health outcome changes, suggesting that immersive engagement in PA may amplify exercise effects even with a relatively brief exposure.

### Comparison to Prior Work

The findings of this study align with and extend a growing body of evidence that immersive VR exercise can enhance motivation, engagement, and health outcomes. Earlier studies demonstrated that immersive and game-based exercise elicits higher enjoyment, perceived competence, and self‑efficacy than traditional exercise and that these qualities translate into greater adherence and positive affect [15,20-22]. More recently, Merola et al [32] reported that a VR high-intensity interval boxing game elicited vigorous heart rate responses while maintaining intrinsic motivation comparable to traditional circuit training, and Boots et al [33] found that active VR games, such as *Beat Saber* and *Gorilla Tag*, produced light to moderate PA levels with high enjoyment among youth. A qualitative study in China showed that young adults with overweight or obesity viewed VR exergames as enjoyable and motivating, although concerns about cost and cybersickness remained [34]. Complementing these findings, a 12-week trial observed that stationary cycling with VR significantly reduced depressive symptoms and produced higher remission rates than non-VR exercise among adults with mild to moderate depression [35]. Likewise, systematic reviews suggest that VR interventions not only alleviate psychological distress but also foster positive mental health [36].

The sustained improvements in motivation and mood observed in this study build upon those acute findings and can be understood through the lens of SDT. The immersive VR environment in our intervention may provide autonomy through user-controlled difficulty and task selection; competence through real-time feedback and visible progress; and relatedness through simulated social features, such as “ghost riders.” Collectively, these elements may have supported basic psychological needs, fostering intrinsic motivation and self-determined exercise behavior; however, this mechanism was not directly measured in this study. Previous studies have shown that when these needs are met, individuals experience enhanced enjoyment, persistence, and well-being [18,19]. The concurrent improvements in intrinsic regulation, vigor, and reduced depressive symptoms observed here likely reflect this motivational pathway. This is consistent with recent findings from Zhang et al [35], who reported that a 12-week VR cycling program significantly reduced depressive symptoms and achieved higher remission rates than non-VR exercise in adults with mild to moderate depression. Together, these results may point to the potential of immersive VR exercise to simultaneously promote mental health and motivation through satisfaction of psychological needs.

From a mental health perspective, our results are consistent with recent evidence indicating that VR interventions not only alleviate psychological distress but also enhance positive functioning. A systematic review of VR interventions for mental health found that immersive environments improve both symptom reduction and well-being by promoting engagement, relaxation, and self-efficacy. The review emphasized that VR-based “positive technologies” can foster emotional resilience and flourishing, aligning with our observed increases in intrinsic motivation and mood [36]. Furthermore, Chen et al [34] found that young adults with overweight or obesity in China viewed VR exergames as highly acceptable and beneficial for motivation and psychological well-being, despite minor concerns about cost or cybersickness. These qualitative insights parallel the high adherence and enjoyment reported in our study, underscoring VR’s ability to overcome motivational barriers commonly faced in traditional exercise.

Physiologically, the improvements in cardiovascular fitness and body composition observed in our study corroborate previous findings that VR exercise can elicit moderate to vigorous intensities comparable to conventional aerobic training. Merola et al [32] observed mean heart rates exceeding 160 bpm during VR boxing, confirming that immersive games can meet intensity thresholds necessary for cardiovascular benefits. Our previous study suggested that VR cycling improved energy expenditure and adherence among university students compared with stationary cycling alone [20,31]. These convergent findings suggest that immersive engagement and attentional distraction during VR exercise may reduce perceived exertion and encourage sustained effort, leading to greater physiological adaptation within shorter time frames [37,38].

Beyond acute fitness outcomes, the integration of VR into PA programs may provide long-term behavioral advantages. Studies have shown that enjoyment and presence in VR are strong predictors of continued participation [39,40]. This aligns with the SDT perspective that affective experiences and perceived autonomy facilitate habit formation and sustained PA behavior. Therefore, immersive VR exercise may not only improve short-term outcomes but also act as a bridge toward more autonomous and lasting engagement in health-promoting activity.

### Limitations

Several limitations should be noted. First, the sample comprised healthy, technology-tolerant college students, which may limit generalizability to less active or older populations. Second, individuals prone to motion sickness were excluded, potentially biasing results toward those more comfortable with VR environments. Third, physiological outcomes were measured using bioelectrical impedance and a submaximal step test, which are less precise than gold-standard laboratory assessments. Fourth, the weekly exercise dose in our intervention (2×60 min; 120 min/week) was below the American College of Sports Medicine recommendation of 150 minutes per week of moderate to vigorous PA; therefore, the observed physiological changes may be overestimated due to less accurate measurements, and the results should be interpreted with caution. Fifth, we used a brief, nonvalidated 2-item screen for cybersickness susceptibility rather than a standardized instrument (eg, Simulator Sickness Questionnaire). This may reduce replicability and limit comparability with previous VR studies. Sixth, the control group did not engage in an active exercise condition, so the contribution of nonspecific effects, such as attention or novelty (Hawthorne effect), cannot be ruled out. In addition, we did not objectively track the control group’s PA during the intervention period, which may limit the certainty that they maintained usual routines and potentially bias the observed changes. Seventh, although the BREQ-2 showed improvements in motivational regulation, we did not measure SDT-based basic psychological needs (ie, autonomy, competence, and relatedness), indicating that the underlying mechanism warrants further investigation. Eighth, this study was conducted during the COVID-19 pandemic and adopted a no-intervention control arm; therefore, we could not fully disentangle the VR-specific effects from the pandemic-related contextual changes. As shown, the control group exhibited elevated depressive symptoms, increased BF percentage, and poorer CRF over time, consistent with real-world COVID-19 pandemic stressors and restricted activity opportunities. Consequently, the observed time×group interactions were likely inflated due to a combination of VR intervention effects and concurrent deterioration in the control group rather than a pure effect against a stable baseline. The findings should be interpreted with caution. Finally, the intervention was delivered in a supervised, in-person laboratory setting, which ensured safety and study fidelity under the COVID-19 pandemic context but might have increased adherence and engagement; consequently, the observed effects might not fully be generalized to unsupervised real-world settings.

### Future Directions

Future research should address several key questions to advance the field of VR-based exercise. First, long-term studies are needed to determine whether the psychological and physiological benefits observed in our 4-week intervention are sustained for months or years and whether VR exercise can promote lasting behavior change and exercise adherence. Second, comparative effectiveness trials with active control groups (eg, traditional cycling and video-guided exercise) are necessary to isolate the unique benefits of VR immersion and determine the added value of VR technology beyond general exercise effects. Third, dose-response studies should investigate the optimal frequency, duration, and intensity of VR exercise sessions for maximizing health benefits while maintaining engagement and minimizing adverse effects such as motion sickness. Fourth, research should explore strategies to make VR exercise more accessible and tolerable for individuals prone to motion sickness, such as gradual exposure protocols or technological improvements. Fifth, studies should examine the effectiveness of VR exercise in more diverse populations, including different age groups, clinical populations, and individuals with varying levels of baseline fitness and technological literacy. Sixth, future studies should adopt validated and objective measures to assess physiological outcomes, such as dual-energy x-ray absorptiometry for body composition and the maximal oxygen uptake test for CRF. Finally, research should investigate the mechanisms underlying the psychological benefits of VR exercise, such as the roles of presence, flow, distraction, and social features, to inform the design of more effective interventions.

### Conclusions

This study indicated that a 4-week immersive VR-based exercise may confer short-term psychological and physiological benefits among college students compared to a no-intervention control group under COVID-19 pandemic–related constraints. However, the supervised context, pandemic-era control deterioration, the untested SDT mechanism, and other design constraints temper causal inference and ecological validity. Future studies should adopt active control designs and be conducted in real-world settings, incorporating objectively determined intensity monitoring and follow-up to further investigate the effectiveness and real-world scalability of VR exercise interventions.

## Appendix

Multimedia Appendix 1 Study questionnaires.

Multimedia Appendix 2 Study recruitment flyer.

Multimedia Appendix 3 Consent form for participation.

Multimedia Appendix 4 CONSORT Checklist. CONSORT: Consolidated Standards of Reporting Trials.

## Abbreviations

BF: body fat
BREQ-2: Behavioral Regulation in Exercise Questionnaire-2
BRUMS: Brunel Mood Scale
CRF: cardiovascular fitness
IPAQ: International Physical Activity Questionnaire
MET: metabolic equivalent
PA: physical activity
SDT: self-determination theory
VR: virtual reality

## Data Availability

The datasets generated or analyzed during this study are available from the corresponding author on reasonable request.
