# Supplementary material for: Effects of an Immersive Virtual Reality–Based Exercise Intervention on Psychological and Physiological Outcomes in College Students: Randomized Controlled Trial
Source: JMIR Serious Games. 2025 Dec 15;13:e75777. doi: 10.2196/75777 (PMC12750068; doi:10.2196/75777)
Supplement: Multimedia Appendix 1 [file games_v13i1e75777_app1.pdf]

PHYSICAL ACTIVITY READYNESS QUESTIONNAIRE (PARQ)

Please answer the following questions by checking “Yes” or “No”.

Questions	Yes	No
1. Has your doctor ever said that you have a heart condition and that you should only perform physical activity recommended by a doctor?		
2. Do you feel pain in your chest when you perform physical activity?		
3. In the past month, have you had chest pain when you were not performing any physical activity?		
4. Do you lose your balance because of dizziness or do you ever lose consciousness?		
5. Do you have a bone or joint problem that could be made worse by a change in<br>your physical activity?		
6. Is your doctor currently prescribing any medication for your blood pressure or<br>for a heart condition?		
7. Do you know of any other reason why you should not engage in physical<br>activity?		

If you have answered “Yes” to one or more of the above questions, consult your physician before engaging in physical activity. Tell your physician which questions you answered “Yes” to. After a medical evaluation, seek advice from your physician on what type of activity is suitable for your current condition.

INTERNATIONAL PHYSICAL ACTIVITY QUESTIONNAIRE (IPAQ)

We are interested in finding out about the kinds of physical activities that people do as part of their everyday lives. The questions will ask you about the time you spent being physically active in the **last 7 days**. Please answer each question even if you do not consider yourself to be an active person. Please think about the activities you do at work, as part of your house and yard work, to get from place to place, and in your spare time for recreation, exercise or sport.

Think about all the **vigorous** activities that you did in the **last 7 days**. **Vigorous** physical activities refer to activities that take hard physical effort and make you breathe much harder than normal. Think *only* about those physical activities that you did for at least 10 minutes at a time.

1. During the **last 7 days**, on how many days did you do **vigorous** physical activities like heavy lifting, digging, aerobics, or fast bicycling?

__________**days per week**

__________No vigorous physical activities → ***Skip to question 3***

2. How much time did you usually spend doing **vigorous** physical activities on one of those days?

__________**hours per day**

__________**minutes per day**

__________Don't Know/Not sure

Think about all the **moderate** activities that you did in the **last 7 days**. **Moderate** activities refer to activities that take moderate physical effort and make you breathe somewhat harder than normal. Think only about those physical activities that you did for at least 10 minutes at a time.

3. During the **last 7 days**, on how many days did you do **moderate** physical activities like carrying light loads, bicycling at a regular pace, or doubles tennis? Do not include walking.

___________**days per week**

___________No moderate physical activities → ***Skip to question 5***

4. How much time did you usually spend doing **moderate** physical activities on one of those days?

__________**hours per day**

__________**minutes per day**

__________Don’t know/Not sure

Think about the time you spent **walking** in the **last 7 days**. This includes at work and at home, walking to travel from place to place, and any other walking that you have done solely for recreation, sport, exercise, or leisure.

5. During the **last 7 days**, on how many days did you **walk** for at least 10 minutes at a time?

__________**days per week**

___________No walking ➡ ***Skip to question 7***

6. How much time did you usually spend **walking** on one of those days?

__________**hours per day**

__________**minutes per day**

__________Don’t know/Not sure

The last question is about the time you spent **sitting** on weekdays during the **last 7 days**. Include time spent at work, at home, while doing course work and during leisure time. This may include time spent sitting at a desk, visiting friends, reading, or sitting or lying down to watch television.

7. During the **last 7 days**, how much time did you spend **sitting** on a **week day**?

__________**hours per day**

__________**minutes per day**

__________Don’t know/Not sure

EXERCISE REGULATIONS QUESTIONNAIRE (BREQ)

Why do you engage in exercise?

We are interested in the reasons underlying peoples' decisions to engage, or not engage in physical exercise. Using the scale below, please indicate to what extent each of the following items is true for you. Please note that there are no right or wrong answers and no trick questions. We simply want to know how you personally feel about exercise. Your responses will be held in confidence and only used for our research purposes.

From 1 to 5, strongly disagree to strongly agree

1.	I exercise because other people say I should	1	2	3	4	5
2.	I feel guilty when I don't exercise	1	2	3	4	5
3.	I value the benefits of exercise	1	2	3	4	5
4.	I exercise because it's fun	1	2	3	4	5
5.	I don't see why I should have to exercise	1	2	3	4	5
6.	I take part in exercise because my friends/family/partner say I should	1	2	3	4	5
7.	I feel ashamed when I miss an exercise session	1	2	3	4	5
8.	It's important to me to exercise regularly	1	2	3	4	5
9.	I can't see why I should bother exercising	1	2	3	4	5
10.	I enjoy my exercise sessions	1	2	3	4	5
11.	I exercise because others will not be pleased with me if I don't	1	2	3	4	5
12.	I don't see the point in exercising	1	2	3	4	5
13.	I feel like a failure when I haven't exercised in a while	1	2	3	4	5
14.	I think it is important to make the effort to exercise regularly	1	2	3	4	5
15.	I find exercise a pleasurable activity	1	2	3	4	5
16.	I feel under pressure from friends/family to exercise	1	2	3	4	5
17.	I get restless if I don't exercise regularly	1	2	3	4	5
18.	I get pleasure and satisfaction from participating in exercise	1	2	3	4	5
19.	I think exercising is a waste of time	1	2	3	4	5

THE BRUNEL MOOD SCALE (BRUMS)

Below is a list of words that describe feelings. Please read each one carefully. Then mark the box that best describes **HOW YOU FEEL RIGHT NOW**. Make sure you answer every question.

		Not at all	A little	Moderately	Quite a bit	Extremely
1.	Panicky					
2.	Lively					
3.	Confused					
4.	Worn out					
5.	Depressed					
6.	Downhearted					
7.	Annoyed					
8.	Exhausted					
9.	Mixed-up					
10.	Sleepy					
11.	Bitter					
12.	Unhappy					
13.	Anxious					
14.	Worried					
15.	Energetic					
16.	Miserable					
17.	Muddled					
18.	Nervous					
19.	Angry					
20.	Active					
21.	Tired					
22.	Bad tempered					
23.	Alert					
24.	Uncertain					

BECK'S DEPRESSION INVENTORY (BDI)

Please circle the scores that best describe you right now.

1.	0	I do not feel sad
	1	I feel sad
	2	I am sad all the time and I can't snap out of it
	3	I am so sad and unhappy that I can't stand it
2.	0	I am not particularly discouraged about the future
	1	I feel discouraged about the future
	2	I feel I have nothing to look forward to
	3	I feel the future is hopeless and that things cannot improve
3.	0	I do not feel like a failure
	1	I feel I have failed more than average person
	2	As I look back on my life, all I can see is a lot of failures
	3	I feel I am a complete failure as a person
4.	0	I get as much satisfaction out of things as I used to
	1	I don't enjoy things the way I used to
	2	I don't get real satisfaction out of anything anymore
	3	I am dissatisfied or bored with everything
5.	0	I don't feel particularly guilty
	1	I feel guilty a good part of time
	2	I feel quite guilty most of time
	3	I feel guilty all of the time
6.	0	I don't feel I am being punished
	1	I feel I may be punished
	2	I expect to be punished
	3	I feel I am being punished
7.	0	I don't feel disappointment in myself
	1	I am disappointed with myself
	2	I am disgusted with myself
	3	I hate myself
8.	0	I don't feel I am any worse than anybody else
	1	I am critical of myself for my weakness or mistakes
	2	I blame myself all the time for my faults
	3	I blame myself for everything bad that happens

9. 0 I don't have any thoughts of killing myself
1 I have thoughts of killing myself, but I would not carry them out
2 I would like to kill myself
3 I would kill myself if I had the chance

10. 0 I don't cry any more than usual
1 I cry more now than I used to
2 I cry all the time now
3 I used to be cry, but now I can't cry even though I want to

11. 0 I am no more irritated by things than I ever was
1 I am slightly more irritated now than usual
2 I am quite annoyed or irritated a good deal of the time
3 I feel irritated all the time

12. 0 I have not lost interest in other people
1 I am less interested in other people than I used to be
2 I have lost most of my interest in other people
3 I have lost all of my interest in other people

13. 0 I make decisions about as well as I ever could
1 I put off making decisions more than I used to
2 I have greater difficulty in making decisions more than I used to
3 I can't make decisions at all anytime

14. 0 I don't feel that I look worse than I used to
1 I am worried that I am looking old or unattractive
2 I feel there are permanent changes in my appearance that make me look unattractive
3 I believe that I look ugly

15. 0 I can work about as well as before
1 It takes an extra effort to get started at doing something
2 I have to push myself very hard to do anything
3 I can't do any work at all

16. 0 I can sleep as well as usual
1 I don't sleep as well as I used to
2 I wake up 1-2 hours earlier than usual and find it hard to get back to sleep
3 I wake up several hours earlier than I used to and cannot get back to sleep

17. 0 I don't get more tired than usual

1 I get tired more easily than I used to
2 I get tired from doing almost anything
3 I am too tired to do anything

18. 0 My appetite is no worse than usual
1 My appetite is not as good as it used to be
2 My appetite is much worse now
3 I have no appetite at all anymore

19. 0 I haven't lost much weight, if any, lately
1 I have lost more than five pounds
2 I have lost more than ten pounds
3 I have lost more than fifteen pounds

20. 0 I am no more worried about my health than usual
1 I am worried about my physical problems like aches, pains, upset stomach
2 I am very worried about physical problems and it's hard to think of much else
3 I am so worried about my physical problems that I cannot think of anything else

21. 0 I have not noticed any recent change in my interest in sex
1 I am less interested in sex than I used to be
2 I have almost no interest in sex
3 I have lost interest in sex completely

Please add up all the scores from the **21** questions.

Total Score ___________________
