# Supplementary material for: Effects of an Immersive Virtual Reality–Based Exercise Intervention on Psychological and Physiological Outcomes in College Students: Randomized Controlled Trial
Source: JMIR Serious Games. 2025 Dec 15;13:e75777. doi: 10.2196/75777 (PMC12750068; doi:10.2196/75777)
Supplement: Multimedia Appendix 2 [file games_v13i1e75777_app2.pdf]

Physical Activity Epidemiology Lab

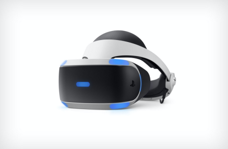

Participation in regular physical activity is vital to a healthy lifestyle. As a college student, however, engaging in a healthy lifestyle can be difficult due to school and work pressures in addition to the increased responsibility brought about by making nutrition and physical activity choices independently for the first time. Luckily, we live in an age of technology wherein virtual reality provides an opportunity for increased physical activity participation. Yet, the effectiveness of virtual reality as a tool for improving health outcomes among college students is largely unstudied.

The purpose of this study is to evaluate the long-term effects of virtual reality-based exercise on college students' physiological and psychological health outcomes.
You will receive $20 incentive gift card upon completion of the study. Think this study is for you? See the participant requirements below.

Participant requirements:

⇒ 18-35 years-old, currently enrolled in the university
⇒ No motion sickness when playing virtual reality-based game or exercise
⇒ Possess no diagnosed severe physical or mental disorder (e.g., cystic fibrosis, multiple sclerosis, schizophrenia, bipolar disorder and major depressive disorder)

Participants will be requested to come into the University of Minnesota's Physical Activity Epidemiology Laboratory for a total of 8 sessions fully immersive virtual reality biking exercise, each session will last 60 minutes, with 2 days per week, for 4 weeks.

Interested? Please contact:

Wenxi Liu
University of Minnesota
Room 310 Williamson hall
231 Pillsbury Drive SE, Minneapolis, MN 55455
Phone: 210-819-9278
Email:
liux4443@umn.edu

Feel free to rip off one of the tabs below if you are in a hurry. We look forward to hearing from you.

Take One!

Virtual Reality Game Study
Wenxi Liu
Liux4443@umn.edu
210-819-9278

Virtual Reality Game Study
Wenxi Liu
Liux4443@umn.edu
210-819-9278

Virtual Reality Game Study
Wenxi Liu
Liux4443@umn.edu
210-819-9278

Virtual Reality Game Study
Wenxi Liu
Liux4443@umn.edu
210-819-9278

Virtual Reality Game Study
Wenxi Liu
Liux4443@umn.edu
210-819-9278

Virtual Reality Game Study
Wenxi Liu
Liux4443@umn.edu
210-819-9278

Virtual Reality Game Study
Wenxi Liu
Liux4443@umn.edu
210-819-9278

Take One!
