# Supplementary material for: Effects of an Immersive Virtual Reality–Based Exercise Intervention on Psychological and Physiological Outcomes in College Students: Randomized Controlled Trial
Source: JMIR Serious Games. 2025 Dec 15;13:e75777. doi: 10.2196/75777 (PMC12750068; doi:10.2196/75777)
Supplement: Multimedia Appendix 3 [file games_v13i1e75777_app3.pdf]

Appendix D. Informed Consent

Title of Research Study: ***Effects of Virtual Reality Exercise on Promoting Physical Activity and Health among College Students: A 4-week Randomized Control Trial***

Investigator Team Contact Information: ***Zan Gao***

For questions about research appointments, the research study, research results, or other concerns, call the study team at:

Investigator Name: Zan Gao<br>Investigator Departmental Affiliation: School of Kinesiology<br>Phone Number: 612-626-4639<br>Email Address: gaoz@umn.edu	Student Investigator Name: **Wenxi Liu**<br>Phone Number: **210-819-9278**<br>Email Address: **liux4443@umn.edu**

Supported By: This research is supported by *Physical Activity Epidemiology Laboratory*.

Key Information About This Research Study

The following is a short summary to help you decide whether or not to be a part of this research study. More detailed information is listed later on in this form.

What is research?

- The goal of research is to learn new things in order to help people in the future. Investigators learn things by following the same plan with a number of participants, so they do not usually make changes to the plan for individual research participants. You, as an individual, may or may not be helped by volunteering for a research study.
- The purpose of this research is to examine the effectiveness of a 4-week immersive-virtual reality (VR) exercise bike intervention on college students' physiological outcomes (physical activity levels, cardiovascular fitness, and body composition) and psychological outcomes (situational motivation, situational interest, mood states, and depressive symptoms).

Why am I being invited to take part in this research study?

We are asking you to take part in this research study because you met the inclusion criteria for participation: 1) 18-35 years old; 2) Possess no diagnosed severe physical or mental disorder; 3) no self-reported motion sickness when playing VR games (e.g., disorientation, dizzy, fatigue, and stomach discomfort).

What should I know about a research study?

- Our research staff will explain this research study to you.
- Whether or not you take part is up to you.
- You can choose not to take part.
- You can agree to take part and later change your mind.
- Your decision will not be held against you.
- You can ask all the questions you want before you decide.

Why is this research being done?

The fact that more than 80% of adults fail to meet the guidelines for both aerobic and muscle-strengthening activities, while the experience of VR is most interesting and appealing to young people. Regrettably, present evidence regarding the use of commercially-available immersive VR headsets and compatible VR exercise apparatus in the promotion of individuals' physiological and psychological health outcomes is lacking. To this end, we will conduct an immersive VR-based exercise intervention exploring the long-term effects of VR-based exercises promoting PA and health-related outcomes among young adults.

How long will the research last?

We expect that you will be in this research study for 4 weeks.

What will I need to do to participate?

You will be asked to engage in a total of 8 VR-based exercise sessions within 4 weeks. You will be asked to exercise on a VR-based exercise bike for 60 minutes each session, and you will be asked to come to exercise twice per week. More detailed information about the study procedures can be found under "What happens if I say yes, I want to be in this research?"

Is there any way that being in this study could be bad for me?

This is no notable health risks regarding the participation of this study. More detailed information about the risks of this study can be found under "*What are the risks of this study? Is there any way being in this study could be bad for me? (Detailed Risks)*"

Will being in this study help me in any way?

More detailed information about the benefits of this study can be found under *"Will being in this study help me in any way? (Detailed Benefits)"*

What happens if I do not want to be in this research?

There are no known alternatives, other than deciding not to participate in this research study.

Detailed Information About This Research Study

The following is more detailed information about this study in addition to the information listed above.

How many people will be studied?

We expect about __40___ people here will be in this research study.

What happens if I say "*Yes, I want to be in this research*"?

If you agree to participate in this study, we will randomly assign you to one of the groups:

- VirZoom virtual reality (VR) exercise bike (intervention group)
- Maintain your usual activities (control group)

Participants will be asked to do the following in addition to engaging in exercise:

- *Anthropometric measurements*: Participant's height and weight measurements will be taken within the University of Minnesota's Physical Activity Epidemiology Laboratory prior to engaging in the 4-week VR exercise intervention.
- *Physical activity levels*: Each participant will be assigned an accelerometer to wear during each 60-minute exercise session. Prior to the start of the intervention, we will ask you to wear an accelerometer for one week for measuring your physical activity levels. In addition, we will ask you to wear the accelerometer for another week at the completion of 4-week intervention.
- *Cardiovascular fitness levels*: we will ask you to step on and off of a 12-inch plyometric box for 3 minutes to the "beep" of a metronome set to 96 beats-per-minute, with each beep corresponding with one leg movement. Immediately following the completion of

the test, the principal investigator will measure participants' heart rate for 60 seconds via palpation of the radial artery on the underbelly of the left wrist.

- Psychological measurements*: we will ask you to complete a series of questionnaires regarding motivation, interest, and mood states to this type of physical activity at baseline and 4 weeks*.

What happens if I say "Yes", but I change my mind later?

You can leave the research study at any time and no one will be upset by your decision. Choosing not to be in this study or to stop being in this study will not result in any penalty to you or loss of benefit to which you are entitled. This means that your choice not to be in this study will not negatively affect your right to any present or future medical care, your academic standing as a student, or your present or future employment.

What are the risks of being in this study? Is there any way being in this study could be bad for me? (Detailed Risks)

The study has the following risks:

(1) Delayed onset muscle soreness occurring several hours to a couple of days after physical activity engagement.

(2) Given the study's potential to increase your physical activity participation a possibility exists for the development of symptoms such as muscular strains/sprains, shortness of breath, joint pain, cramping, fatigue, fainting, and dizziness.

(3) Although very unlikely, mental distress caused by the administration of assessments of physical activity-related psychosocial constructs is possible.

(4) There is a potential risk for unidentified heart issues during exercise that may impact you.

Will it cost me anything to participate in this research study?

There will be no costs to you as a result of taking part in this study other than the time spent participating. Taking part in this study is voluntary.

Will being in this study help me in any way? (Detailed Benefits)

During the participation of this study, your weekly time spending in physical activity may increase, also, this study offers you an opportunity to exercise under a fully immersive virtual environment, and you may find it enjoyable to exercise while playing virtual reality games.

What happens to the information collected for the research?

The records of this study will be kept private. Your record for the study may, however, be reviewed by designated departments at the University with appropriate regulatory oversight. We will not include any information in publications or presentations that will make it possible to identify you. To these extents, confidentiality is not absolute. Study data will be encrypted according to current University policy for protection of confidentiality.

Will I receive research test results?

The investigator(s) will not contact you or share your individual test results. However, the summary of the research findings may be available upon request.

Will anyone besides the study team be at my consent meeting?

No. There will be only the study team at your consent meeting.

Whom do I contact if I have questions, concerns or feedback about my experience?

This research has been reviewed and approved by an IRB within the Human Research Protections Program (HRPP). To share feedback privately with the HRPP about your research experience, call the Research Participants' Advocate Line at 612-625-1650 (Toll Free: 1-888-224-8636) or go to z.umn.edu/participants. You are encouraged to contact the HRPP if:

- Your questions, concerns, or complaints are not being answered by the research team.
- You cannot reach the research team.
- You want to talk to someone besides the research team.
- You have questions about your rights as a research participant.
- You want to get information or provide input about this research.

Will I have a chance to provide feedback after the study is over?

The HRPP may ask you to complete a survey that asks about your experience as a research participant. You do not have to complete the survey if you do not want to. If you do choose to complete the survey, your responses will be anonymous.

If you are not asked to complete a survey, but you would like to share feedback, please contact the study team or the HRPP. See the "Investigator Contact Information" of this form for study team contact information and "Whom do I contact if I have questions, concerns or feedback about my experience?" of this form for HRPP contact information.

What happens if I am injured while participating in this research?

In the event that this research activity results in an injury, treatment will be available,

including first aid, emergency treatment and follow-up care as needed. Care for such injuries will be billed in the ordinary manner, to you or your insurance company. If you think that you have suffered a research related injury let the study physicians know right away.

Will I be compensated for my participation?

If you agree to take part in this research study, we will pay you _$20.00_____ for your time and effort.

Your signature documents your permission to take part in this research. You will be provided a copy of this signed document.

____ ___________________

Signature of Participant Date

____

Printed Name of Participant

____ ___________________

Signature of Person Obtaining Consent Date

____

Printed Name of Person Obtaining Consent
